# Consumer Perceptions of Home-Based Percussive Massage Therapy for Musculoskeletal Concerns: Inductive Thematic Qualitative Analysis

**DOI:** 10.2196/52328

**Published:** 2024-02-05

**Authors:** Saloni Butala, Pearl Valentine Galido, Benjamin K P Woo

**Affiliations:** 1 College of Osteopathic Medicine of the Pacific Western University of Health Sciences Pomona, CA United States; 2 Chinese American Health Promotion Laboratory University of California, Los Angeles Los Angeles, CA United States

**Keywords:** home-based therapy, injury prevention, massage guns, musculoskeletal pain, pain management, percussive massage therapy, rehabilitation, self-management, sports medicine

## Abstract

**Background:**

Musculoskeletal pain is a prevalent concern among diverse populations, from the average individual to the elite athlete. Handheld percussive massage therapy devices like massage guns have gained much popularity in both medical and athletic settings. Its application has been prominently recognized in injury prevention and rehabilitation. The expansion of the market to provide handheld percussive therapy devices with varying features and price points has encouraged professional and novice use. While percussive therapy holds similarities to more studied therapeutic modalities, like vibration therapy and soft tissue mobilization, there is limited evidence-based information on the indications and contraindications.

**Objective:**

This study aims to use a qualitative analysis of consumer perceptions to understand the perceived therapeutic potential of percussive massage therapy as a home-based intervention for musculoskeletal concerns of everyday users and elite athletes. Additionally, we aim to gain insight on valuable characteristics supporting its therapeutic potential as well as pertinent limitations.

**Methods:**

The TOLOCO massage gun (TOLOCO) was identified as the best-selling percussive massage therapy device on Amazon. We performed an inductive thematic qualitative analysis on the top 100 positive comments and the top 100 critical comments of the device between June 2020 and April 2023 to determine 4 relevant themes.

**Results:**

The 4 themes identified upon qualitative analysis were pain management, versatility, accessibility, and safety and user education. Consumer reviews indicated use for this percussive therapy device in adolescents, adults, and older people across a spectrum of activity levels. Consumers reported the therapeutic potential of percussive massage therapy in managing wide-ranging musculoskeletal concerns like acute pain, chronic pain, nonsurgical injury rehabilitation, postsurgical injury rehabilitation, and injury prevention. Consumers highlighted the versatility of the device to address person-specific needs as a key feature in supporting its perceived therapeutic benefits. Additionally, consumers frequently commented on the affordability and availability of this device to increase accessibility to home-based care. Some critical reviews emphasized a concern for the quality of the device itself. However, this concern did not translate to the overall modality of percussive massage therapy. Of note, despite strong approval for its therapeutic potential, consumer reviews lacked evidence-based insights on appropriate usage.

**Conclusions:**

Home-based percussive massage therapy holds value with its perceived efficacy in pain management for acute and chronic conditions, as well as in injury prevention and rehabilitation. As a low-cost and readily available device for everyday users and high-performing athletes, percussive massage therapy works toward establishing increased health care accessibility and optimizing health care usage. This home-based intervention can serve to reduce the significant personal and economic burden of prevalent musculoskeletal concerns. However, the limited scientific research on percussive massage therapy raises concerns about the lack of evidence-based care and indicates the need for future studies.

## Introduction

Musculoskeletal pain can significantly impact the physical and mental well-being of a wide range of individuals [1,2]. Not only that, but musculoskeletal pain has also been shown to increase the economic burden on both the individual and the health care system [3-5]. Thus, handheld percussive massage therapy has continued to gain popularity for its application in musculoskeletal pain, injury prevention, and recovery in both the medical and athletic realms. The use of this therapeutic mechanism transitioned beyond the office setting to home due to a vast array of manufacturing companies like Therabody and Hyperice. Now, Amazon’s platform offers a wide variety of percussive massage therapy devices with different features and price points. In increasing accessibility to percussive massage therapy, Amazon opens the market for professional and novice use.

Percussive therapy is said to have originated in the mid-20th century by Robert Fulford and involves the delivery of high-velocity and low-amplitude oscillating forces to the body [6]. It is proposed to be a notable method of myofascial release [7]. The myofascial system works by distributing tension across a network of connective tissue covering muscles, bones, and organs [8,9]. Due to the continuity of this system, tissue overload or repetitive strain injuries in one region of the body can create dysfunctional biomechanics, impairments in functional movement patterns, and referred tension in other regions of the body [8]. In acting as a myofascial release modality, percussive massage therapy can potentially serve to renew the fascial tissues and manage their restrictive distortions.

Percussive therapy is suggested to incorporate components of more well-studied therapy modalities like vibration therapy and conventional massage [10]. Vibration therapy is said to elicit its therapeutic impact on muscle fibers and proprioception, with health outcomes demonstrating improvements in elasticity, mobility, lymphatic and blood circulation, and swelling [11]. Soft tissue, a common modality of conventional massage, has shown similar health benefits with regard to improvements in circulation, range of motion, and muscle relaxation [12,13]. Its suggested mechanism of action involves reducing friction between fascial layers, improving muscle fiber patterns, and reducing the buildup of abnormal hyaluronic acid molecules in implicated regions [13]. In combining these approaches, percussive therapy has been postulated to promote biomechanical and molecular functioning by improving circulation and lymphatic flow, increasing range of motion, and reducing pain perception and adhesions [8,10]. There are limitations present in detailing the physiological mechanism of percussive therapy itself, given the lack of current evidence-based research.

Today, percussive massage therapy is widely used and has the capacity to mimic conventional therapeutic approaches by serving as a possible self-myofascial release modality. This paper primarily aims to analyze consumer perceptions of the massage gun, a well-known percussive massage therapy modality, in order to gain further insight on its therapeutic potential as a home-based intervention for musculoskeletal concerns of both the everyday user and high-performing athletes. Additionally, this paper seeks to gather information on valued characteristics that support its therapeutic potential as well as pertinent limitations that comment on its necessity for improvement.

## Methods

### Study Design

This study used an inductive thematic qualitative analysis to explore consumer perceptions of the therapeutic potential and limitations of home-based percussive massage therapy. Qualitative analysis has been deemed a suitable methodology for drawing insights and perspectives from the human experience [14,15]. The authors used an inductive thematic framework to derive data-driven insights and perspectives on this topic without predetermined input [16].

### Data Source

Through Amazon’s search engine, the TOLOCO massage gun was identified as the best-selling handheld massage gun on Amazon. Given the consumer trend toward web-based shopping platforms in combination with Amazon’s diverse market and large influence in e-commerce, the authors found Amazon to be an appropriate data source for consumer reviews [17-19]. The authors performed a qualitative analysis on consumer reviews of this device between June 2020 and April 2023 to interpret consumer perceptions of home-based percussive massage therapy [17,19,20].

### Data Collection

The inclusion criteria for this qualitative analysis required the consumer review to be a verified purchase by Amazon, fall between the June 2020 and April 2023 time frame, and include a written review alongside its rating. The authors applied the indicated inclusion criteria to 35,985 total ratings and 7516 verified purchase consumer reviews. The top 100 positive and top 100 critical comments of this subset were used for analysis [17,19,20]. Positive and critical categories were predetermined by Amazon itself. A total of 4 positive consumer comments were discarded as the content was categorized incorrectly or lacked a formal review. Additionally, 2 critical comments were discarded as the content was categorized incorrectly or was incomprehensible. Reviews written in languages apart from English were translated through Amazon’s translate feature. Data was stored on a cloud-based platform. All information was deidentified before data storage and use. Consumer reviews were left unedited for authenticity.

### Data Analysis

An inductive thematic qualitative analysis was performed on consumer reviews of the TOLOCO massage gun on Amazon. On initial analysis of consumer reviews, the authors manually developed a codebook based on pertinent key points and common patterns [16]. Some code examples included: “muscle recovery,” “postsurgical care,” “accessories,” “multiple modes,” “price point,” “self-therapy,” “user manual,” “battery defect,” and “longevity.” After the development of this initial codebook, a secondary analysis was conducted to ensure appropriate coding adjustments for all transcripts. The final codebook consisted of a total of 40 codes. After completion of coding, an analysis was carried out on the codebook itself in order to derive 4 distinct themes of percussive massage therapy. Subthemes of the 4 overarching themes were also generated. For example, the theme “accessibility” included subthemes of “affordability” and “availability.” The authors performed a third review of consumer transcripts and applied the relevant identified themes and subthemes to each [15]. Quotes that were found to best represent each theme and subtheme were used to better illustrate consumer perceptions of percussive massage therapy.

### Ethical Considerations

The data for this qualitative analysis were gathered from publicly available information. Thus, this research was deemed exempt from the University of California, Los Angeles institutional review board. This study does not qualify as human subjects research and therefore does not require further informed consent or compensation. All public data were deidentified before use. Generative artificial intelligence was not used in the context of this paper.

## Results

### Findings

The TOLOCO massage gun had a 4.5-star rating with 35,985 total ratings and 7516 verified purchase consumer reviews. Of those 7516 reviews, there were 5936 positive reviews and 1580 critical reviews. In analyzing the top 100 positive reviews and the top 100 critical reviews, 4 pertinent themes were identified: pain management, accessibility, versatility, and safety and user education. Consumer demographics such as age, gender, and location were not readily available unless specified within the consumer review itself.

### Theme 1: Pain Management

Pain management was one of the most common positive indications for this device based on consumer reviews, with 51 positive comments discussing some form of therapeutic purpose. Under pain management, consumer reviews suggested the handheld percussive therapy device be adapted to a diversity of patient circumstances, including daily pain, chronic pain, nonsurgical injury management, postsurgical injury management, and injury prevention. Table 1 details information derived from both positive and critical reviews for each category encompassing pain management.

**Table 1:** Consumer perceptions of percussive massage therapy in pain management.

Type of pain	Consumer perceptions
Acute aches and pains	I can’t wait for my next Charley horse calf spasm. I am going to jab this gun at max setting into that contacting calf muscle and turn it into tenderized sirloin. Also works well at blasting away muscle knots and tensions in my trapezius area. I am a side sleeper, spend long hours at desk and driving which causes problems. This blasts away deep tissue knots and tension away. I sleep better and wake up with greater range of motion.
Chronic pain	I used to be a black diamond skier in my youth, and unfortunately, all those young and reckless checks that I wrote when I was younger are being cashed now. I wish I had a time machine so I could go back and tell that idiot how much arthritis I would have when I got older because of the crazy stunts I pulled. Anyway, to wrap things up I absolutely 100 percent strongly recommend these percussion guns to help with all kinds of aches and pains.
Nonsurgical injury rehabilitation	I have been seeing massage therapists for several months to work on a strained muscle and my T-band and a tight psoas muscle. She used this device to help break up the huge knot in my leg that she and another therapist have been working on for several months. This massage gun did the trick! So, I bought one to have at home as I gently start exercising my leg muscles again.
Postsurgical injury rehabilitation	Just had a hip replacement and the muscles in my leg and hip knotted up. Got this bad boy and wacked my leg and hip till I couldn’t stand it anymore 3 days later it was gone.
Injury prevention	I’m training for a 10K and I know my legs are going to be sore and I’m excited to have this to help manage that over the next few months.

Some consumers mentioned being introduced to this percussive therapy device by health care providers. This prompted consumers to conduct their own research to identify the appropriate at-home percussive therapy device to best meet their health needs. One positive review stated:

A couple of months ago, I didn’t even know massage guns existed. Enter a physical therapist who used one of these on my leg during a session. I was so impressed by how much it helped that I started doing some research and found this gun.

In analyzing the top 100 critical reviews, 17 commented on the product’s capacity to contribute beneficially toward their pain management regimen. A total of 10 consumers felt dissatisfied with the product’s pain management capabilities. Despite the number of critical reviews analyzed, only 1 indicated that percussive massage therapy devices were overall not the best product for them. In critical reviews, common input suggested this particular device was either lacking in intensity or too powerful—issues that might be mitigated by more cushioned attachments, improved quality, or a similar device by a different manufacturer.

### Theme 2: Versatility

Versatility was the second-most commonly discussed key feature of the TOLOCO massage gun. Consumers discussed its various applications supported by the 15 attachment heads and speed adjustability. These various attachments allow for targeted massage of different muscle groups. Both the speed adjustability and the many attachments gave consumers the opportunity to personalize their user experience. Not only that, but consumer experiences also highlighted how the versatility of this percussive therapy device reaches varying populations, age groups, and person-specific needs. For instance, a consumer discussed its benefits for different needs in their own household:

We have a household full of athletes, and the gun proved invaluable in losing up knots and returning blood flow to aching muscles. Our daughter (a dancer), used in two to three times a week, and I would use it after 4+ hour rides (bicycling).

Overall, consumers appeared to agree that the versatility of this device contributed to its therapeutic potential.

### Theme 3: Accessibility

Of the top 100 positive reviews, 29 consumers discussed accessibility as a key feature of this handheld percussive therapy device. Accessibility was referenced when consumer reviews discussed aspects of either affordability or availability. Regarding affordability, a consumer commented:

I was recommended by my physiotherapist to use a percussion massage gun to loosen up my calves and shoulders, but I couldn’t justify paying $300 or more for a Theragun.

Another consumer even compared this budget-friendly model to those seemingly more expensive and stated:

I have the Theragun mini and this gun outperforms the Theragun big time. Waaaay quieter, feels smoother, the attachments are game changing. I was nervous due to the low price, but so far it’s been light years better than my Theragun and light years cheaper.

While not all consumers agreed with this statement, most positive reviews suggested it to be a quality product for its price point.

Most critical reviews commented on the longevity of the device, with consumers stating that they experienced battery or internal defects resulting in product malfunction. In this case, some consumers indicated that they opted for a product replacement as they found percussive therapy to be a cost-effective and ideal method of managing musculoskeletal pain. For instance, a consumer stated*:*

After a couple weeks it started making a high-pitched humming sound. Amazon was fantastic about sending me a replacement very quickly. It’s too bad because these are priced very well, and they seem to be built well.

Other consumers returned the device because they found the overall quality to be a point of concern.

### Theme 4: Safety and User Education

The final identified theme upon qualitative analysis was safety and user education. While not a direct component of the therapeutic potential and limitations of the device, it is a notable mention to establish better practices for its use. This comprised guidance from both the manufacturer and other consumers as well as established safety features. Consumer recommendations were based on personal knowledge, experiences, or errors, as demonstrated in Table 2. However, no consumer reviews commented on the use of direct evidence-based guidelines to facilitate usage of this device.

**Table 2:** Consumer-based recommendations regarding safety and education of percussive massage therapy.

Consumer experience	Consumer recommendations
Personal knowledge	I would advise doing some due diligence and research to make sure you get the right model and brand for you that suits your needs. I also recommend starting slow, with shorter sessions so you don’t overdo it. Muscles can release some chemicals (lactic and Uric acid) during massage and if they aren’t used to these it could make you a little sore at first. It’s like going to a chiropractor. At first it can feel worse, then it feels better. Same kind of thing here but it can be mitigated with a slower titration of time spent daily on massage.
User experience	Caution is in order when working around thin muscle tissue near bone and joints, since the 12 mm travel distance of the TOLOCO Massage Gun can cause discomfort quickly if there is insufficient tissue depth to lessen the impact on hard structures.
User error	It would not charge but I finally figured out that I grabbed the wrong charger. So user error, it is still working great now.

Consumer reviews indicated the product manufacturer established safety components, including a user manual and an automatic shutdown feature. Consumers found the user manual to assist in appropriate and optimal product usage by indicating detailed information on how to use the device and its fifteen attachments. The automatic shutdown feature of the TOLOCO massage gun turned off the device after 10 minutes of use to protect both users and the device. A consumer commented:

Speaking of the 10-minute limit, both devices recommend limiting your sessions to 10 minutes at each sitting. This is partly due to physiological reasons and to prevent you from overworking your muscles. But also, you need to give the motor on these kinds of devices some rest to prevent overheating. The Toloco has an auto-shut off function that turns the device off after 10 minutes of continuous use. I appreciate this feature as it protects my device.

In critical reviews, however, a consumer noted their frustration with the 10-minute automatic shutdown feature and stated:

The biggest thing about it that bugs me is that it shuts off every 10 mins or so and requires me to turn it back on again. This shouldn’t be a problem for people using it less than that, but I use it for an hour at a time and it gets really annoying having to turn it on over and over again.

## Discussion

### Overview

This qualitative analysis of consumer perceptions of the TOLOCO massage gun on Amazon commented on the generalized therapeutic potential of handheld percussive massage therapy. While this paper focused on an individual percussive therapy device and comments on specific features of said device, this qualitative analysis served to gain insight on the therapeutic potential and limitations of using generalized percussive massage therapy as a home-based intervention for musculoskeletal concerns. The qualitative analysis of consumer reviews demonstrated use for this device in adolescents, adults, and older people. Its use was displayed across a spectrum of activity levels, ranging from bedridden to sedentary to high-performing athletes. In analyzing the top 100 positive and top 100 critical verified purchase reviews, 4 pertinent themes were identified: pain management, versatility, accessibility, and safety and user education. Both positive and critical consumer reviews suggested this percussive therapy device addressed wide-ranging musculoskeletal concerns, including daily pain, chronic pain, nonsurgical injury management, postsurgical injury management, and injury prevention. Critical reviews regarding the device’s pain management capacity were primarily regarding device-specific features and suggested identifying an alternative percussive therapy device. The critical reviews highlighted the variability of personal preferences or needs rather than the generalized inability of percussive massage devices to have a therapeutic function. Positive consumer reviews emphasized the budget-friendly nature of the TOLOCO massage gun as a key feature in improving accessibility to the device and therefore its therapeutic potential. However, critical consumer reviews commented on the concern for product quality at lower price points in comparison to their more expensive counterparts. Regarding the final theme, safety and user education, consumer reviews demonstrated this aspect through product-specific safety features, manufacturer manuals, and peer-to-peer guidance. While not directly commented on by consumers, it is apparent that no consumer mentioned evidence-based guidelines for facilitating the use of this device.

Musculoskeletal pain, whether acute or chronic, is a common complaint in the health care system [21,22]. Such pain increases in prevalence with aging and lifestyle factors, such as occupation or lack of physical activity [22]. Both the personal and economic burden of musculoskeletal pain have been demonstrated globally across diverse populations [2,22-24]. For instance, the increasing presence of work-induced musculoskeletal pain in individuals without preexisting conditions has been discussed among nurses, postal workers, agricultural workers, and office workers [24-27]. One can suggest that this concept be readily translated to alternate occupations that also involve long working hours and significant lifting, standing, or sitting, thus being applicable to a vast majority of individuals. Occupation-related musculoskeletal pain is a pertinent common thread among the average individual and is one example that directly increases health care usage and expenditures [2,22]. Not only that, but also such pain increases both absenteeism and presenteeism and therefore negatively impacts employers financially [2]. For the individual, work-related musculoskeletal pain significantly impacts quality of life both physically and mentally [22]. This emphasizes the need to identify appropriate intervention modalities, particularly in the realm of home-based care.

While the mechanism of percussive therapy at the molecular level has not been well defined, its plausible application and health outcomes have been demonstrated by various studies. For athletes, percussive therapy has been found to improve muscle endurance and delay muscle fatigue without compromising muscle performance [10,28]. Some studies have also commented on the capacity of percussive therapy to improve explosive muscle strength, a valuable dynamic for athletes, while other studies claim no significant association in this domain [6,29]. The benefits of percussive therapy can impact everyday users in addition to high-performing athletes. The everyday user may include individuals from ageing or working populations as well as those with orthopedic needs. For instance, working populations—whether involving extended computer usage, long standing hours, or heavy physical labor—experience increasing strain on the body [30-32]. Initially, this strain may present as acute aches and pains, but repeated exposure can increase the potential for greater chronicity of pain [30-32]. Percussive massage therapy can serve as an adequate home-based musculoskeletal pain intervention for the everyday user. In reducing stiffness, increasing muscle relaxation, and improving muscle tone, percussive therapy encourages the flexibility of muscles and tendons and therefore establishes a better range of motion [6,29,33-35]. Not only that, but also in reducing the tension of muscles and tendons, it additionally works to alleviate perception of pain and thus yield psychosocial benefits [6,29]. The TOLOCO massage gun demonstrated the capacity of percussive massage therapy to be an easily accessible therapeutic modality that is readily available within one’s own home. Thus, it gives users the opportunity to take their health into their own hands as well as augment medical rehabilitation for improved health outcomes. A breadth of users found this percussive massage therapy device to be a resourceful tool in their pain management regimens. Additionally, some consumers discussed alternative uses for the device in the context of myalgias secondary to chemotherapy, menstrual pains, and migraines.

It is imperative that we consider the biopsychosocial approach to care when addressing musculoskeletal pain. Acute or chronic pain is recognized as a contributing factor to an individual’s mental health [36-39]. Pain post orthopedic intervention can be associated with long-term disability, increased restrictions in work or daily living, and decreased satisfaction overall [39]. These points of association may explain elevated depression rating scores in this population [39]. Alternately, in considering a population of high-performing athletes dedicated to their athletic identity and role in sport, it is apparent that there is an association between sport-related injury and mental health [36-38,40,41]. Over the course of 5 academic years, from 2009-2010 to 2013-2014, over 1 million injuries were estimated within the National Collegiate Athletic Association [42]. Given the rising competition and pressure, it is likely that this number has continued to rise. Sport-related injuries both short-term and long-term, negatively impact current and previous elite athletes [36-38,40]. In understanding the interconnectedness between musculoskeletal pain and perceived stress, anxiety, and depression, it is crucial that we analyze possible points of intervention. For instance, the psychological aspect of chronic low back pain may encompass decreased self-efficacy and autonomy [43]. Home-based percussive massage therapy, when appropriate, can possibly serve to encourage patient connectedness to care and increase patient confidence in caring for themselves. This concept highlights the potential of home-based percussive therapy as one branch of biopsychosocial interventions in patient care.

A total of 2 prominent handheld percussive massage therapy devices on the market are the Theragun by Therabody and the Hypervolt by Hyperice. Both products tend to range between US $100 and US $500—a price range that might not be considered affordable by all. As the therapeutic percussive therapy device continued to gain popularity, numerous manufacturers such as TOLOCO joined the expanding market to produce low-cost products and therefore improve its accessibility. In comparison to the listed prices of the more well-known brands, TOLOCO lists its product at approximately US $50 and intermittently includes discounted prices or coupons. The expansion of this market to include low-cost items is important because, by improving the affordability of these products, one can say it simultaneously increases health care accessibility. Not only that, but also this accessibility allows numerous consumers of varying backgrounds to find personal therapeutic purposes in the device. Additionally, it is imperative to recognize how the burden of musculoskeletal pain disproportionately impacts low-income populations [44-47]. While social determinants of health impede one’s ability to access comprehensive care, it also increases an individual’s risk to such conditions and amplifies the burden of disease [45-48]. The gaps in accessing health care providers, psychosocial support, and health resources perpetuate the disparities experienced by these communities and have negative implications for health outcomes [46,48]. Therefore, creating cost-effective, home-based interventions for musculoskeletal ailments may act as a therapeutic modality for wide-ranging populations and serve as one plausible method of bridging the gap between the health care system and vulnerable populations.

While this percussive massage therapy device has specific safety and user education components, including a user manual and automatic shutdown feature, it is imperative to further evaluate the safety and efficacy of such unregulated, at-home modalities. Previous case reports have discussed the consequences of inappropriate usage of these devices, which include rhabdomyolysis, vertebral artery dissection, and lens dislocation alongside secondary acute angle-closure glaucoma [49-51]. When considering at-home therapy options such as percussive massage therapy, users may not have essential medical knowledge and therefore may be unaware of certain anatomical structures including tissue, bones, and vasculature. They may also not fully understand the possible interaction of this percussive modality with their own underlying conditions. Of note, one user of the TOLOCO massage gun commented on their frustration with the 10-minute automatic shutdown and discussed disregarding the safety feature in place. This highlights the possibility of a lack of user education as well as unregulated use of the device. The authors of the noted case reports equally advocated for detailed evaluation of the safety of such devices in order to better define guidelines for indications and contraindications [49-51]. More comprehensive user education of at-home percussive massage therapy may dissuade such inappropriate usage and consequential traumatic complications while contributing to greater beneficial impacts.

### Limitations

This qualitative analysis was conducted on consumer perceptions of a single percussive massage therapy device, despite the abundance of such products on the market. The TOLOCO massage gun was selected based on statistics suggesting that this particular product was the best-selling on Amazon. However, this does not indicate that it is the best-selling percussive massage therapy device in the current expanded market. The variability of these products with regard to their affordability, additional features, longevity, and programming may contribute to consumer perceptions. With this analysis primarily investigating this product from the perspective of serving as a home-based therapeutic modality, it might be implied that not all users have the same background knowledge and understanding of how to use the device optimally. Given that this was a retrospective study on consumer reviews, this analysis only includes perceptions from a snapshot in time from individuals who were willing to comment on the capabilities of and concerns about the product’s usage. In using public data for this qualitative analysis, the authors were unable to facilitate further conversation, gain clarification, or identify sociodemographic characteristics among consumers. Additionally, this analysis does not provide objective or longitudinal data to further define indications or contraindications for percussive massage therapy. Though percussive massage therapy devices are deemed quite popular and beneficial based on consumer perceptions, there is currently limited scientific research available on their underlying physiologic mechanisms. Thus, further exploration with regard to its safety and efficacy is imperative.

### Future Research

Previous studies have been conducted on the possible effects of percussive massage therapy. A study demonstrated that localized vibrations induced by massage guns at 38 Hz and 47 Hz can increase circulation to the region and therefore aid in the muscle recovery of healthy young athletes [52]. In the strength and conditioning setting, the use of massage guns allowed for increased muscle strength and explosive muscle performance secondary to delayed fatigue while also reducing musculoskeletal pain perception [6,10,28,53]. Another study using ultrasound diagnostics found that the use of massage guns on the thoracolumbar fascia resulted in a reduction in echo intensity in that region due to the movement of hyaluronic acid toward the fascial rim and thus improved lubrication and gliding between fascial layers [54]. While these studies have demonstrated the possible effects of percussive massage therapy and postulated potential reasons for these effects, they were unable to conclusively define the physiologic mechanisms. A study surveyed health care professionals about their perceptions and use of massage guns; however, it also emphasized the lack of current evidence-based guidelines [33,55]. Thus, future research is needed to investigate the underlying mechanisms of percussive massage therapy to better outline its safety and efficacy. Second, this research may create better guidelines to optimize care for different populations and prevent at-home users from sustaining further injury. In the future, it may be valuable to conduct further research on the integration and cost-effectiveness of mobile health apps and sensing technology in conjunction with home-based percussive therapy devices.

### Conclusions

Handheld percussive massage therapy devices such as the TOLOCO massage gun hold potential value as a home-based therapeutic modality. The qualitative analysis of consumer perceptions revealed 4 pertinent themes: pain management, versatility, accessibility, and safety and education. Per consumer insight, percussive massage therapy was shown to address pain management for wide-ranging musculoskeletal needs in diverse populations. In providing an opportunity for consumers—from elite athletes to the everyday user—to play an active role in their own health, handheld percussive massage therapy can navigate the intersection of physical and mental well-being and thus encompass a biopsychosocial approach to care. Additionally, this home-based intervention has the potential to work toward addressing the significant economic burden of musculoskeletal pain by reducing and optimizing health care usage and expenditures. In considering the diversity of user needs and circumstances, this at-home modality addressed a pertinent health care concern in that it improved accessibility to care by presenting as both an affordable and readily available device for consumers. The variability of device models with low-cost price points introduces a possible platform for health equity in this domain of care. While this provides users with the opportunity to essentially play a larger role in their own care, future research on the safety and efficacy of home-based percussive massage therapy is imperative and can ultimately serve to promote evidence-based guidelines, further its technological development, and expand its therapeutic potential.

## Data Availability

Data sharing is not applicable to this article as no data sets were generated or analyzed during this study.
